# Determination of cardiovascular risk in 192,711 underweight and normal weight workers

**DOI:** 10.55730/1300-0144.5392

**Published:** 2022-02-27

**Authors:** Bárbara ALTISENCH JANÉ, José Ignacio RAMÍREZ-MANENT, Sebastiana ARROYO BOTE, Pere RIUTORD SBERT, Hilda María GONZÁLEZ SAN MIGUEL, Ángel Arturo LÓPEZ GONZÁLEZ

**Affiliations:** 1 Balearic Islands Health Service, Balearic Islands, Spain; 2 University School ADEMA Palma, Balearic Islands, Spain

**Keywords:** Cardiovascular risk, body mass index (BMI), weight

## Abstract

**Background/aim:**

***:*** The aim of this study was to determine the prevalence of cardiovascular risk factors (CVRF) and the level of cardiovascular risk (CVR), determined with different scales (REGICOR, SCORE, ERICE, vascular age...) in people with low and normal weight.

**Materials and Methods:**

A total of 192,711 underweight and normal weight Spanish workers participated. CVR parameters included were weight, abdominal circumference, blood pressure, glycemia (high >125 mg/dL or under hypoglycemic treatment) and lipids (cut-off points: total cholesterol 200 mg/dL, LDL 130 mg/dL, triglycerides 150 mg/dL) were obtained by automated enzymatic methods. Body mass index (BMI) was calculated, considering underweight 18.5 and normal weight 18.5–24.9. A descriptive analysis of the categorical variables was carried out.

**Results:**

The values of analytical, anthropometric and clinical parameters were more unfavorable in the normal weight group. Also, the prevalence of alterated values of CVR seen with different scales show higher risk in this group and in all cases the values in men are worse. It was seen that the variable with the greatest influence on the appearance of altered values of the cardiovascular risk scales (CVRS), influencing all of them was the age.

**Conclusion:**

***:*** All the CVRS analyzed showed higher values in the group of people with normal weight compared to those with underweight. Age, followed by sex, were the variables that most influence the appearance of high CVR values.

## 1. Background/aim

The CVR of any population is directly related to the prevalence of the different risk factors and to the impact that each of them may have on morbidity and mortality due to cardiovascular disease (CVD).

Obesity, classically understood as a body mass index greater than 30 kg/m2, is considered a chronic and multifactorial pathology [1,2]. The World Health Organization (WHO) defines obesity as excessive body fat accumulation, which is associated with several risks to health [2]. In recent decades, it has reached epidemic proportions, with serious social and psychological consequences, affecting all socioeconomic groups and all ages [3]. Many factors have led to this uncontrolled growth in obesity, including the increase in the consumption of high-calorie foods [4,5], the increase in sedentary lifestyles due to a change in entertainment patterns, mainly due to the replacement of leisure time related to physical activity by sedentary activities (television, electronic devices) [6,7]. Many studies have already linked obesity to an increase in cardiovascular mortality and mortality from other causes [8]. It is also known that obesity is a CVRF in itself [9,10].

Just as we have seen that the relationship between excess weight and CVD or risk has been extensively studied, the level of CVR in people with body mass index (BMI) below 25 kg/m^2^, and especially below 18.5 kg/m^2^, i.e. people with low and normal weight, is not as well known. The BMI is used as an indicator of excess body fat, but it only provides an approximation of body fat and does not reveal fat distribution. Furthermore, the same parameters are used for the worldwide population, without making differences in some risk factors that can be important when estimating CVR [11]. Sometimes, individuals can be misclassified as not being at risk or over classified as being at risk without having it.

For this reason, the aim of this study was to determine the prevalence of CVRFs and the level of CVR, determined with different scales such as REGICOR, SCORE, ERICE and vascular age, in people with low and normal weight.

## 2. Materials and methods

A cross-sectional study was carried out in a group of 192,711 underweight and normal weight workers from different Spanish geographic areas between January 2019 and July 2020. Workers were selected from among those who went to periodic occupational health medical examinations considering the following inclusion criteria:

- Be actively working.
- Accepted their voluntary participation in the study and authorized the use of data for epidemiological purposes.
- No previous serious CVD (myocardial infarction, cerebrovascular disease, etc.).
- BMI under 25 kg/m^2^.
- The Figure is a representative flowchart of participants included in the study.

The different anthropometric, analytical and clinical determinations were performed by health professionals from the occupational health units participating in the study, after homogenizing the measurement techniques.

### 1.1. Cardiovascular risk parameters included in the assessment

Weight (in kg) and height (in cm) were measured with a SECA 700 measuring rod and a SECA 220 telescopic measuring rod with millimeter division.

Abdominal circumference (in cm) was determined with a SECA 200 tape measure and with the person in a standing position and the abdomen relaxed, placing the tape measure at the height of the last floating rib.

Blood pressure was measured with a calibrated OMRON M3 automatic sphygmomanometer after 10 min rest. The mean of three measurements was obtained. We considered hypertension to be values of 140 mm Hg systolic or 90 mm Hg diastolic. Blood tests were obtained by peripheral venipuncture after fasting for 12 h.

Glycemia, total cholesterol and triglycerides were obtained by automated enzymatic methods, HDL were determined by precipitation with dextran sulfate MgCl_2_, and LDL (mg/dl) were calculated by Friedewald formula (provided triglycerides were <400 mg/dLl).

Friedewald formula: LDL = total cholesterol − HDL − triglycerides / 5.

All values are expressed in mg/dL. Glycemia was considered high if the values were greater than 125 mg/dL or if the patient was under hypoglycemic treatment [12]. The following cut-off points are established for lipids: total cholesterol 200 mg/dL, LDL 130 mg/dL and triglycerides 150 mg/dL. BMI was calculated by dividing weight by height in squared meters. Underweight was considered under 18.5 and normal weight between 18.5 and 24.9.

REGICOR (adaptation of the Framingham scale to the Spanish population) [13] estimates the risk of suffering a cerebrovascular event over a 10-year period. It is applied between 35 and 74 years of age. The risk is moderate at 5% and high at 10% or higher [14].

The SCORE scale applied in the Spanish population [15,16] estimates the risk of suffering a fatal cerebrovascular event over a 10-year period. It is applied between 40 and 65 years of age. The risk is moderate in >4% and high in >5%. Calibrated tables were used to determine vascular age [17].

Vascular age with the Framingham model [17] can be calculated in persons over 30 years of age, whereas vascular age with the SCORE model [18] between 40 and 65 years. An interesting concept applicable to both vascular ages is ALLY (avoidable years of life lost) [19], which can be defined as the difference between biological age (BA) and vascular age (VA).


ALLY=VA-BA.

A person is considered to be a smoker if he/she has regularly consumed one or more cigarettes per day (or the equivalent in other types of consumption) during the last month, or if he/she has quit smoking less than 12 months ago.

Social class is determined from the 2011 National Classification of Occupations (CNO-11) according to the proposal of the Spanish Society of Epidemiology [20]. Three social classes are differentiated: I. Directors/managers, university professionals, athletes and artists. II. Intermediate occupations and self-employed workers without employees. III. Unskilled workers.

### 2.2 Statistical analysis

A descriptive analysis of the categorical variables was carried out, calculating the frequency and distribution of responses for each of them. For quantitative variables, the mean and standard deviation were calculated, and for qualitative variables the percentage was calculated. A bivariate association analysis was performed using the χ^2^ test (with a correction with the Fisher’s exact test, when conditions required so) and a Student’s t-test for independent samples. For the multivariate analysis, binary logistic regression was used with the Wald method, with an odds ratio calculation and a Hosmer–Lemeshow goodness-of-fit test. Statistical analysis was performed with the SPSS 27.0 program, and a p value of <0.05 was considered as statistically significant.

### 2.3 Ethical considerations and aspects

The study was approved by the Clinical Research Ethics Committee of the Health Area of Balearic Islands in November 2020. All procedures were performed in accordance with the ethical standards of the institutional research committee and with the 2013 Declaration of Helsinki. All patients signed written informed consent documents prior to participation in the study.

## 3. Results

The mean values of analytical, anthropometric and clinical parameters, in both sexes, are more unfavorable in the normal weight group than in the underweight group. In all cases, the values in men are worse than in women. The prevalence of smoking is higher in women and in underweight individuals. All the data are presented in Table 1.

The CVRS analyzed show higher values in the normal weight group in both sexes, although the differences are only statistically significant in the case of vascular age with the Framingham model. All the values, as we have seen above, are more unfavorable among men. The complete data are shown in Table 2.

The prevalence of altered values of scales related to CVR follows the same trend seen with the mean values, that is, the worst results are found in the normal weight group. The prevalences are also higher in men. The differences are only statistically significant for hypertension and lipid profile. The complete data can be found in Table 3.

Multivariate analysis by binary logistic regression with the Wald method included age 50 years or older, smoking, male sex, and social class III as covariates. Age was the variable with the greatest influence on the appearance of altered values of the CVRS, influencing all of them. Male sex was the second most influential variable, affecting all the scales except elevated LDL cholesterol. Belonging to the normal weight group increased the risk compared to the underweight group in all scales except diabetes. The least influential variable was tobacco consumption. The complete data set is presented in Table 4.

## 4. Discussion

It is known that there are various parameters that influence the appearance of high CVR and therefore the development of acute and chronic diseases that increase morbidity and mortality in the general population [1,2,9,10]. The lifestyle of the current world population, with a tendency to sedentary and an unhealthy lifestyle, has increased the risk of developing pathologies [21].

Most of the studies that refer to CVR are carried out in populations with overweight and/or obesity and there are few studies referring to our research topic, carried out in a population with normal weight or underweight.

In our study, all the CVRS analyzed showed unfavorable values in the group of people with normal weight compared to those with underweight. Age, followed by sex, are the variables that most influence the appearance of high CVR values. Social class and, to a lesser extent, weight gain also seem to have an influence. That is why healthy lifestyle changes are important to prevent future cardiometabolic alterations that cause an increase in BMI and therefore a worsening of the parameters that affect CVR, such as increased blood pressure, fat mass percentage, the increased abdominal and waist circumference, as well as a worsening of laboratory parameters that refer to blood glucose and lipid profile [22,23].

Unfortunately, we have not found articles similar to ours that assess the level of CVR in this specific group, so we cannot directly compare our results with those obtained previously by other authors since most of the studies that appear in the literature compare the changes in different CVRF between a population with normal weight and with obesity or overweight, but not between normal weight and underweight population. This fact will allow our work to be considered as a reference in this population and may serve as a starting point for future research.

The study carried out by McEniery et al. [24], carried out in 2511 persons aged between 18 and 40 years, free of CVD and medication, in which the factors underlying hypertension were assessed in normal weight versus obese individuals showed that there were different mechanisms involved in hypertension in both groups, so the individuals with normal weight also had altered blood pressure levels, something that has also been objectified in our study. In the obese patients, the main mechanism was the increase in cardiac output due to their larger body size. On the other side, in normal weight individuals, the most important mechanism was the increase in peripheral vascular resistance, and it was also observed that BMI values were higher in hypertensive individuals. Similarly, in our study, it was demonstrated in a statistically significant way that individuals with normal weight had altered values in most of the parameters related to CVR, so that even if the population are within the normal weight range, those with a higher BMI will present worse results in the different parameters analyzed as can be seen in the results of our study.

The DORICA study, carried out by Millán et al. [25], analyzed the prevalence of the different risk factors and established that the most appropriate cut-off points to establish a high CVR were BMI of 27 kg/m2 in women and 30 kg/m2 in men. According to the World Health Organization, they would be within the range of overweight and obesity, so this study concludes that the higher the BMI, the greater the risk of presenting and adverse event. Therefore, referring to our study, those participants with a higher BMI, despite being within the normal weight range, also had a high CVR compared to underweight patients, where their BMI was lower.

Other studies, such as the one carried out by Berg et al. [11] or the study by Correa-Rodriguez et al. [22], or the one by Elagizi et al. [26], evaluated the alterations and CVR in people with normal weight, but who were considered obese since they had an excess of body fat [26]. This group, despite having a normal weight BMI, the CVR was higher in those with a higher percentage of body fat [22]. They also observed that when comparing the level of CVR between people of normal weight (with and without excess body fat), it was observed that those with excess fat had higher triglyceride and LDL levels and lower HDL levels, as well as higher blood pressure values [11].

To conclude, although most of the studies found in the literature indicate that obese or overweight individuals have a greater risk of developing adverse events due to the increased CVR. The data provided in our study show that populations with normal weight may also have altered different parameters that influence CVR and therefore have it increased, having a normal BMI, which indicates that being in normal weight is not synonymous with the absence of risk. That is why it is important to maintain a healthy lifestyle, with adequate nutrition, daily physical activity and avoid sedentary lifestyle in order to reduce CVRFs.

The strengths of the study include the very large sample size, almost 200,000 persons, the large number of CVRS analyzed (REGICOR, CVR SCORE, ERICE, Framingham vascular age and SCORE vascular age) and the fact that it is one of the few studies to have assessed CVR in persons with low weight or normal weight without obesity. Most of the articles found in the literature study CVR in normal weight obese or in obese or overweight individuals.

Its limitations are that it was carried out in a specific geographical area and in a working population, which might limit the generalizability of our results to other areas and to the general population.

## Figures and Tables

**Figure:**
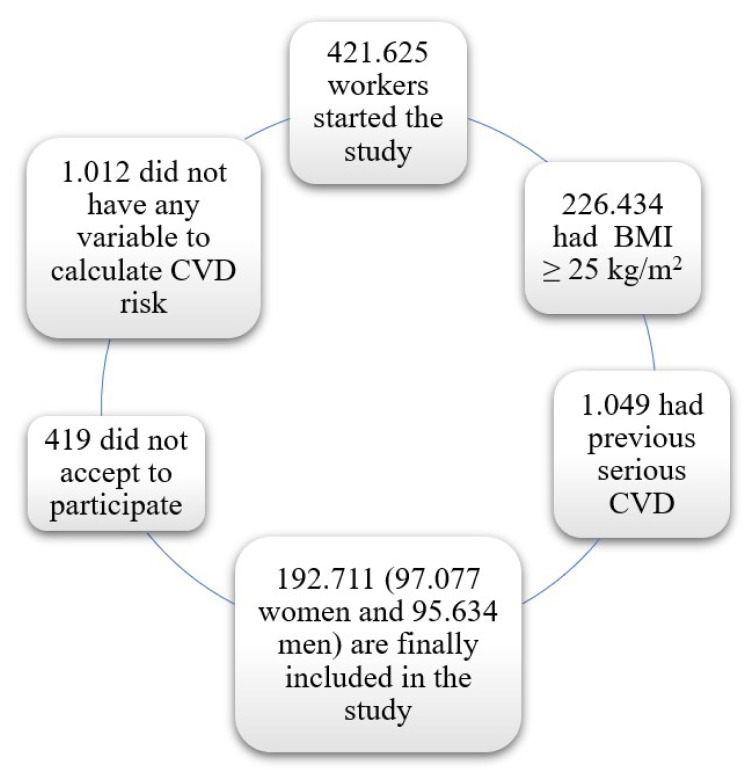
Flowchart of participants. BMI: body mass index, CVD: cardiovascular disease, KG: kilogram, m: meter

**Table 1:** Sociodemographic, anthropometric, clinical and analytical characteristics of the sample.

	Underweight	Normal weight		Underweight	Normal weight	
	Women n = 5768	Women n = 91,309		Men n = 2448	Men n = 93,186	
	Mean (SD)	Mean (SD)	p-value	Mean (SD)	Mean (SD)	p-value
**Age**	33.05 (9.75)	37.92 (10.43)	<0.0001	30.93 (11.05)	37.14 (10.92)	<0.0001
**Height**	163.49 (6.37)	162.37 (6.35)	<0.0001	175.91 (7.09)	175.16 (6.94)	0.124
**Weight**	47.20 (4.17)	58.18 (6.20)	<0.0001	54.67 (4.94)	70.00 (7.34)	<0.0001
**Waist**	63.93 (5.10)	69.56 (6.04)	<0.0001	70.26 (7.46)	78.58 (7.32)	<0.0001
**SBP**	108.62 (13.00)	113.80 (13.94)	<0.0001	117.96 (13.78)	123.40 (13.80)	<0.0001
**DBP**	67.67 (9.00)	70.29 (9.52)	<0.0001	70.34 (9.60)	73.84 (9.87)	<0.0001
**Cholesterol**	175.20 (32.50)	186.50 (34.55)	<0.0001	164.53 (33.53)	182.90 (37.08)	<0.0001
**HDL-c**	60.04 (8.63)	58.94 (8.55)	<0.0001	54.34 (6.79)	53.58 (7.73)	0.221
**LDL-c**	100.35 (31.89)	111.76 (33.92)	<0.0001	93.36 (33.22)	109.72 (35.54)	<0.0001
**Triglycerides**	74.07 (29.71)	79.13 (35.95)	<0.0001	85.01 (53.82)	99.10 (64.51)	<0.0001
**Glycaemia**	83.56 (10.96)	85.29 (12.22)	<0.0001	86.77 (19.19)	89.07 (17.49)	<0.0001
	**Percentage**	**Percentage**	**p-value**	**Percentage**	**Percentage**	**p-value**
**18–29 years**	42.30	24.50	<0.0001	55.43	28.55	<0.0001
**30–39 years**	32.68	32.38		23.37	31.02	
**40–49 years**	18.15	27.94		12.34	25.17	
**50–70 years**	6.87	15.18		8.86	15.26	
**Social class I**	8.08	8.46	<0.0001	1.51	5.17	<0.0001
**Social class II**	24.20	26.81		5.92	14.70	
**Social class III**	67.72	64.73		92.57	80.13	
**Nonsmoker**	64.60	66.90	<0.0001	64.95	65.78	0.202
**Smoker**	35.40	33.10		35.05	34.22	

DBO: diastolic blood pressure; HDL-c: high density lipoproteins – cholesterol; LDL-c: low density lipoproteins – cholesterol; SBP: systolic blood pressure.

**Table 2:** Mean values of cardiovascular risk scales in underweight and normal weight by sex.

		Underweight		Normal weight			Underweight		Normal weight	
		Men		Men			Women		Women	
	n	Mean (SD)	n	Mean (SD)	p-value	n	Mean (SD)	n	Mean (SD)	p-value
**ALLY vascular age SCORE**	518	6.08 (6.78)	37571	6.13 (6.48)	0.876	1436	2.54 (4.51)	39277	3.41 (4.94)	0.078
**SCORE scale**	518	1.34 (1.79)	37571	1.36 (1.95)	0.913	1436	0.23 (0.81)	39277	0.33 (0.81)	0.887
**ALLY vascular age Framingham**	1091	0.46 (8.23)	66565	2.79 (8.66)	<0.0001	3325	–4.73 (7.62)	68914	–2.41 (9.68)	0.031
**REGICOR scale**	759	2.47 (1.76)	52194	2.75 (1.84)	0.124	2246	1.46 (1.21)	54453	1.84 (1.59)	0.221
**ERICE scale**	1091	2.91 (3.29)	66558	3.39 (3.99)	0.223	3325	1.73 (2.29)	68899	2.22 (2.76)	0.287

**Table 3:** Prevalence of alterated values of cardiovascular risk scales in underweight and normal weight by sex.

		Underweight		Normal weight			Underweight		Normal weight	
		Women n = 5768		Women n = 91,309			Men n = 2448		Men n = 93,186	
	n	Percentage	n	Percentage	p-value	n	Percentage	n	Percentage	p-value
**Hypertension**	5768	3.48	91309	7.34	<0.0001	2448	8.21	93186	15.85	<0.0001
**Cholesterol ≥200 mg/dL**	5768	20.11	91309	32.05	<0.0001	2448	14.3	93186	30.17	<0.0001
**LDL-c ≥130 mg/dL**	5768	16.33	91309	27.03	<0.0001	2448	13.6	93186	27.15	<0.0001
**Triglycerides ≥150 mg/dL**	5768	2.46	91309	3.97	<0.0001	2448	6.62	93186	12.03	<0.0001
**Glycaemia 100–125 mg/dL**	5768	4.16	91309	6.16	0.310	2448	9.03	93186	12.24	0.112
**Glycaemia >125 mg/dL**	5768	0.29	91309	0.50		2448	1.92	93186	1.56	
**SCORE moderate**	1436	1.88	39277	2.52	0.479	518	12.36	37571	12.35	0.775
**SCORE high**	1436	0.28	39277	0.82		518	8.69	37571	7.64	
**REGICOR moderate**	2246	3.12	54453	6.23	0.098	759	9.99	52194	12.63	0.117
**REGICOR high-very high**	2246	0.09	54453	0.41		759	0.92	52194	0.91	
**ERICE moderate**	3325	0.84	68899	1.58	0.084	1091	5.13	66558	5.87	0.339
**ERICE high-very high**	3325	0.12	68899	0.07		1091	0.27	66558	1.13	

**Table 4:** Binary logistic regression.

	≥ 50 years		Men		Normal weight		Social class III		Smokers	
	OR (95% CI)	p-value	OR (95% CI)	p-value	OR (95% CI)	p-value	OR (95% CI)	p-value	OR (95% CI)	p-value
**Hypertension**	4.33 (4.19–4.47)	<0.0001	2.45 (2.37–2.53)	<0.0001	1.89 (1.70–2.09)	<0.0001	1.22 (1.14–1.32)	<0.0001		ns
**Cholesterol ≥200 mg/dL**	4.01 (3.91–4.12)	<0.0001	0.92 (0.90–0.94)	<0.0001	1.84 (1.74–1.95)	<0.0001	1.15 (1.10–1.20)	<0.0001		ns
**LDL-c ≥130 mg/dL**	4.08 (3.98–4.19)	<0.0001		ns	1.82 (1.71–1.93)	<0.0001	1.14 (1.09–1.20)	<0.0001		ns
**Triglycerides ≥150 mg/dL**	2.05 (1.97–2.13)	<0.0001	3.23 (3.11–3.36)	<0.0001	1.71 (1.52–1.92)	<0.0001	1.28 (1.17–1.40)	<0.0001	0.95 (0.92–0.99)	0.009
**Glycaemia >125 mg/dL**	4.79 (4.38–5.24)	<0.0001	3.05 (2.74–3.39)	<0.0001		ns	1.91 (1.68–2.18)	<0.0001		ns
**SCORE moderate-high**	100.56 (90.30–111.98)	<0.0001	14.57 (13.53–15.70)	<0.0001	1.28 (1.02–1.61)	0.035	1.28 (1.18–1.39)	<0.0001	7.81 (7.31–8.35)	<0.0001
**REGICOR moderate-high-very high**	22.50 (21.31–23.77)	<0.0001	2.47 (2.36–2.59)	<0.0001	1.76 (1.46–2.11)	<0.0001	1.49 (1.40–1.59)	<0.0001	3.48 (3.32–3.64)	<0.0001
**ERICE moderate-high-very high**	61.32 (48.27–74.68)	<0.0001	5.62 (5.24–6.03)	<0.0001	1.27 (1.01–1.62)	0.042	1.26 (1.16–1.38)	<0.0001	2.62 (2.45–2.78)	<0.0001
